# Sex-specific associations between the environmental exposures and low-grade inflammation and increased blood pressure in young, healthy subjects

**DOI:** 10.1038/s41598-024-59078-4

**Published:** 2024-04-26

**Authors:** Wojciech M. Marchewka, Krzysztof L. Bryniarski, Jakub M. Marchewka, Iwona Popiołek, Grzegorz Dębski, Rafał Badacz, Ida Marchewka, Natalia Podolec-Szczepara, Barbara Jasiewicz-Honkisz, Tomasz P. Mikołajczyk, Tomasz J. Guzik

**Affiliations:** 1 Department of Internal and Agricultural Medicine, Faculty of Medicine, Jagiellonian University Medical College, Skarbowa 1, 31-121 Krakow, Poland; 2 Department of Radiology and Imaging Science, 5th Military Hospital, Krakow, Poland; 3 Department of Interventional Cardiology, Institute of Cardiology, Jagiellonian University Medical College, Krakow, Poland; 4 Department of Cardiology, Thoraxcenter, Erasmus University Medical Center, Rotterdam, The Netherlands; 5 Department of Physiotherapy, University of Physical Education, Krakow, Poland; 6 Department of Orthopedics and Trauma Surgery, 5th Military Hospital, Krakow, Poland; 7 Department of Toxicology and Environmental Diseases, Jagiellonian University Medical College, Krakow, Poland; 8 Department of Ophthalmology, Ludwik Rydygier Memorial Specialized Hospital, Krakow, Poland; 9 Pediatrics Cardiology Department, Jagiellonian University Medical College, Krakow, Poland; 10 Center for Medical Genomics OMICRON, Jagiellonian University Medical College, Krakow, Poland; 11 BHF Centre for Research Excellence, Centre for Cardiovascular Sciences, The University of Edinburgh, Edinburgh, UK

**Keywords:** Air pollution, Noise pollution, Blood pressure, Systemic inflammation, Hs-CRP, Homocysteine, Cardiovascular diseases, Immunological disorders, Environmental impact

## Abstract

Long-term exposures to environmental factors including airborne as well as noise pollutants, are associated with cardiovascular risk. However, the influence of environmental pollution on the young population is controversial. Accordingly, we aimed to investigate the relationships between long-term exposures to different environmental factors and major cardiovascular and inflammatory parameters and biomarkers in young, healthy subjects. Representative sample of permanent residents of two cities differing in air and noise pollution levels, aged 15–21 years, were recruited. Krakow and Lublin, both located in southern Poland, were chosen in relation to their similarities in demographic and geopolitical characteristics, but differences in air pollution (higher in Krakow) and noise parameters (higher in Lublin). A total of 576 subjects were studied: 292 in Krakow and 284 in Lublin. All subjects underwent health questionnaire, blood pressure measurements and biomarker determinations. Inflammatory biomarkers, such as CRP, hs-CRP, fibrinogen as well as homocysteine were all significantly higher in subjects living in Krakow as opposed to subjects living in Lublin (for hsCRP: 0.52 (0.32–0.98) mg/l vs. 0.35 (0.22–0.67) mg/l; *p* < 0.001). Increased inflammatory biomarker levels were observed in Krakow in both male and female young adults. Interestingly, significant differences were observed in blood pressure between male and female subjects. Males from Krakow had significantly higher mean systolic blood pressure (127.7 ± 10.4 mm/Hg vs. 122.4 ± 13.0 mm/Hg; *p* = 0.001), pulse pressure (58.7 ± 8.9 mm/Hg vs. 51.4 ± 12.3 mm/Hg; *p* < 0.001) and lower heart rate (*p* < 0.001) as compared to males living in Lublin. This was not observed in young adult females. Long-term exposure to environmental factors related to the place of residence can significantly influence inflammatory and cardiovascular parameters, even in young individuals. Interestingly, among otherwise healthy young adults, blood pressure differences exhibited significant variations based on biological sex.

## Introduction

Air pollution (AP), noise as well as socioeconomic status are risk factors for cardiovascular diseases^1–4^.While most of the environmental factors are interrelated, the majority of the studies focused only on one of them neglecting the potential influence of the others such as noise, soil and water pollution. According to Lancet Commission, the environmental pollutants are responsible for around 9 million premature deaths worldwide^5^. Air pollution is the single largest environmental cause of disease and it was responsible for 6.7 million premature deaths in 2019^5^. Long term exposure to air pollution influences human health and life expectancy^6^. This is probably because ambient air pollution is independent risk factor of development and progression of pulmonary and cardiovascular diseases, and especially hypertension^7–9^. However, other factors which usually coexist with air pollution in urban areas such as noise exposure should be taken into the account as they can also influence both the development and progression of cardiovascular diseases. It has been shown that noise pollution increases blood pressure^10,11^. Moreover, according to the recent study, the traffic related noise pollution increases the risk of development of ischemic heart disease^12^. The link between AP and systemic inflammation can be mediated by macrophages and epithelial cells which stimulate production of acute phase proteins such as C—reactive protein (CRP)^13,14^. Similarly, the chronic noise exposure may activate autonomous nervous system and endocrine signaling, which then stimulate the inflammatory response^15^. Although the exact mechanisms of both environmental factors are not fully understood, it has been suggested that the long-term exposure to ambient air pollution and noise pollution is associated with the systemic inflammatory response resulting in vascular dysfunction, accelerated atherosclerosis and hypertension^16–18^. Moreover, previous studies provided the evidence of the association of high sensitive CRP (hs-CRP) with major cardiovascular risk^19^. Disorders caused by the air pollution can develop over time^8,16^. Moreover, elevated blood pressure in young people is positively correlated with the prevalence of hypertension and cardiovascular events in adults, in the future^20,21^.

Very few studies assessed the impact of different environmental factors on cardiovascular as well as systemic inflammation amongst population of young subjects. Moreover, most of these studies investigated the single environmental factor while many of these factors are known to act together to cause both the inflammation and cardiovascular complications, leading to inconsistent results and conclusions.

Accordingly, we aimed to examine the potential link between long-term exposure to environmental factors such as air pollution and noise, and their impact on systemic inflammation and cardiovascular parameters, including blood pressure, in healthy young individuals who are permanent residents of two cities, Krakow and Lublin, which have generally similar characteristics but vary in environmental pollution levels.

## Materials and methods

### Population

**Figure 1:**
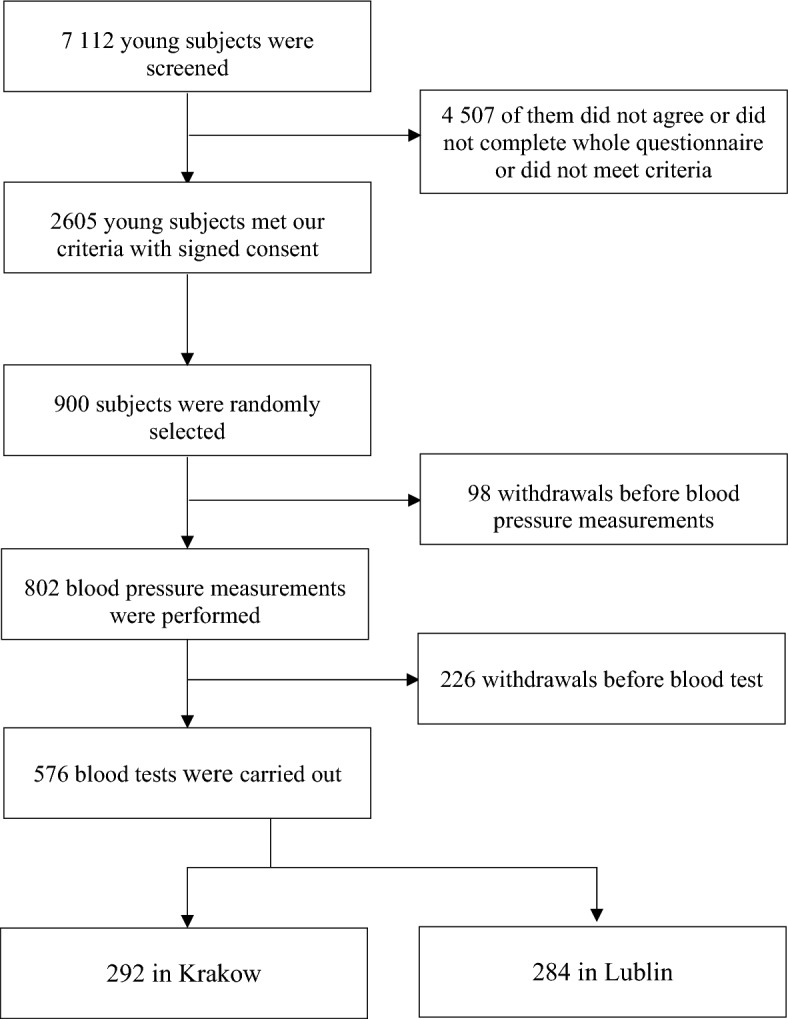
Flow chart of the study population.

The study was conducted in 2014 from January to April. We recruited young, healthy, Caucasian subjects from secondary schools and universities in Krakow and Lublin. Each participants fulfilled questionnaire including data on inter alia: age, sex, weight, height, residency, education, smoking and comorbidities. Inclusion criteria were as follows: (1) age between 15 and 21 years; (2) residents of Krakow or Lublin, respectively for at least 10 years continuously. Exclusion criteria were as follows: (1) diagnosed cardiovascular disease; (2) symptoms of acute infection; (3) diabetes mellitus; (4) diagnosed chronic hormonal abnormalities; (5) history of neoplasm; (6) lack of written consent and (7) smoking cigarettes or other products. Both cohorts had similar socioeconomic status. Detailed flow chart is shown in Fig. 1.

### Ethics declaration

The study was approved by the Jagiellonian University Bioethics Committee in Krakow, Poland (Approval No: KBET/113/B/2013). Written informed consent was obtained from all the participants before enrolment. In the case of minors, informed consent from their parents was obtained first. All procedures were performed in accordance with the World Medical Association Declaration of Helsinki and its later amendments.

### Blood pressure measurements

After completing questionnaires 900 subjects were randomly selected and underwent blood pressure and heart rate measurements that were performed according to guidelines of European Society of Hypertension^22^. We used clinically validated blood pressure monitors Omron M3 Comfort. Blood pressure measurements were performed by qualified medical personnel between 9 and 11 AM in medical centers where also blood samples were collected.

### Inflammatory biomarkers and other blood tests

Furthermore, venous blood samples were collected from participants both living in Krakow and in Lublin. We measured complete blood count, CRP, hs-CRP, homocysteine, fibrinogen, glucose, cholesterol, low density lipoprotein (LDL), high density lipoprotein (HDL), triglycerides, thyroid-stimulating hormone (TSH) and uric acid.

Qualified nurse collected in summary 20 ml of fasting blood from the cubital vein into three separate vacuum blood collection tubes. It was performed in external company medical centers in Krakow and Lublin between 9 and 11 AM, the same day as the blood pressure measurements. We used the same measuring conditions in each place. All surveys and procedures were supervised by the same team members. Complete blood count was performed with the Sysmex XN 2000 Automated Blood Cell Analyzer. Other blood parameters were examined with the Cobas 6000 analyzer.

### Environmental data

We extracted detailed air and noise pollutant data from the database of Inspectorate for Environmental Protection (2003–2013). The pollution monitoring stations were situated in corresponding districts of the cities; however, exact locations varied. The detailed data together with summary of pollution levels are published periodically at Regional Inspection for Environmental Protection website^23^.

#### Statistical analysis

Sample size was calculated based on previous studies available. Continuous variables are presented as mean ± standard deviation (SD) or median and interquartile range (Q1–Q3), depending on the normality of distribution. The normality of distribution was tested using the Shapiro–Wilk test. Qualitative variables were analyzed by enumerating the count and percentage occurrence of each value. Qualitative variables in groups were compared using the chi-squared test with Yates correction for 2 × 2 tables or with Fisher’s exact test. Intergroup comparisons were analyzed using t- test for independent samples, or Mann Whitney U test. The correlations between quantitative variables were assessed using Spearman’s correlation coefficient. All *p*—values are two-sided, *p* < 0.05 was considered statistically significant. Calculations were performed using Statistica 13.3 (TIBCO Software Inc. USA).

## Results

### Participant characteristics

A total of 576 young, healthy subjects (382 females, 194 males) were recruited among pupils and students, 292 from Krakow and 284 from Lublin. There were no differences between groups in age and body mass index (BMI), as shown in Table 1. These two populations differed in terms of sex distribution. There were significantly more females in Lublin in comparison to Krakow (Table 1).

**Table 1:** Basic characteristic of the study groups.

Parameter	Krakow (n = 292)	Lublin (n = 284)	*p*
Age, years (mean ± SD)	17.9 ± 0.9	18 ± 1.1	0.56
Sex, female n (%)	176 (60.27%)	206 (72.54%)	**0.002**
BMI (kg/m^2^)	21.52 ± 2.91	21.39 ± 2.96	0.45
Smoking, n (%)	0 (0%)	0 (0%)	–
Parental smoking, n (%)	157 (53.77%)	130 (45.77%)	0.09
Diagnosed CVD, n (%)	0 (0%)	0 (0%)	–
Acute infection, n (%)	0 (0%)	0 (0%)	–

Values are presented as mean ± standard deviation (SD) or n (%).

BMI—body mass index, CVD—cardiovascular disease.

*p*—values are indicated.

Significant values are in bold.

**Figure 2:**
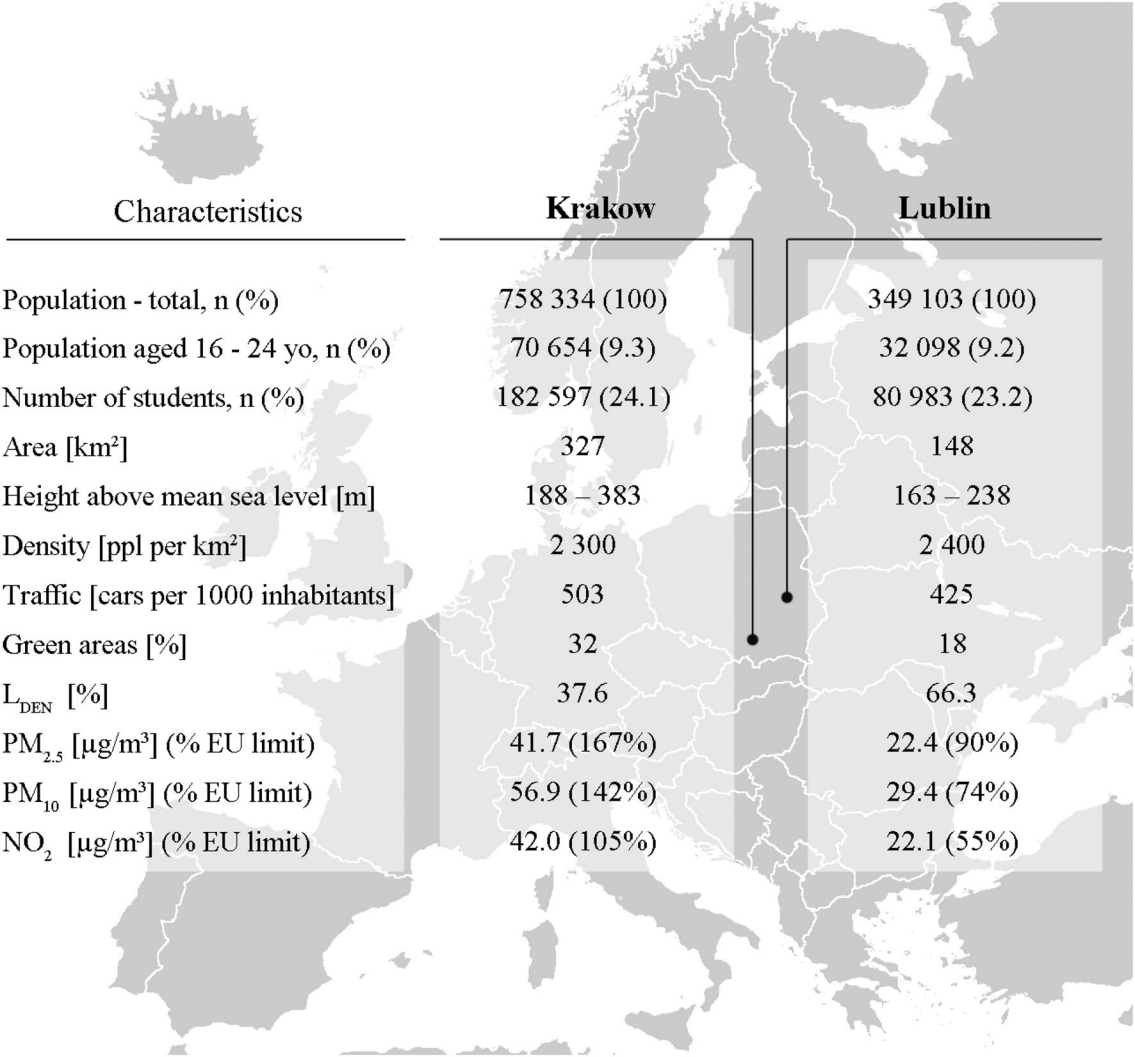
Characteristics of the demographic, geographical and environmental data of Krakow and Lublin in 2013, both urban areas. Mean air quality values for selected air pollutants during 2003–2013 in Krakow and Lublin compared with air quality limit values for air pollutants under EU legislation, LDEN—day–evening–night noise level index, PM—particulate matter, EU—European Union.

We chose Lublin and Krakow as two comparable cities with significant differences in environmental pollution levels. Both cities are capitals of the regions, with similar densities and medical research centers. Detailed characteristics of these cities showing the main similarities (Fig. 2).

The level of air pollution is different in Krakow and Lublin (Fig. 2). Krakow and surrounding Malopolska region are the most polluted Polish areas, and one of the most polluted region in Europe^7^. In Krakow, as a major urban hub, the high population density and extensive road network lead to significant emissions from vehicles, exacerbating air pollution, especially in congested areas. Conversely, Lublin experiences lower traffic volumes, yet still contends with emissions from various vehicles. Situated in the Lublin Upland, Lublin’s relatively flat terrain contrasts with Krakow’s diverse topography, potentially influencing air circulation and pollutant dispersion. These differences underscore the need for tailored pollution mitigation strategies in each city, considering their distinct transportation dynamics and geographical characteristics. There are the differences in particulate matter (PM) and nitrogen dioxide levels between cities^23^. Average levels of PM_2.5_, PM_10_ and NO_2_ in Krakow were far exceeding EU Air Quality Directives: Directive 2008/50/EC of the European Parliament and of the European Council on ambient air quality and cleaner air for Europe^24^. Lublin is significantly less polluted city than Krakow with average AP levels below EU limits. Mean air quality values for studied air pollutants in Krakow and Lublin are presented in the Fig. 2. However, in case of noise pollution described with 24-h average sound levels, known as the day–evening–night noise level (L_DEN_) index, Krakow had better results than Lublin, which means that more Lublin inhabitants were subjected to noise pollution. Regarding water pollution both cities comply with EU norms according to 98/83/EC of 3 November 1988 Council directive.

### Cardiovascular parameters

In the next step, we wanted to examine whether participants recruited in Krakow and Lublin differ in terms of the main cardiovascular parameters such as systolic blood pressure (SBP), diastolic blood pressure (DBP), pulse pressure (PP) and heart rate (HR). We found significantly higher heart rate (HR) among Lublin participants (Table 2). This was particularly observed in males. Moreover, we observed higher systolic blood pressure among Krakow subjects, however these results were not statistically significant. We did not observe any differences in diastolic blood pressure between Krakow and Lublin cohorts. Interestingly, males from Krakow had significantly higher mean systolic blood pressure, pulse pressure and lower heart rate as compared to males living in Lublin (Table 2). Furthermore, we observed significantly higher pulse pressure (PP) and SBP among female participants from Lublin (Table 2).

**Table 2:** Blood pressure, heart rate, and pulse pressure in male and female participants from Krakow and Lublin.

Parameter	Female			Male			Total		
	Krakow n = 176	Lublin n = 206	*p*	Krakow n = 116	Lublin n = 78	*p*	Krakow n = 292	Lublin n = 284	*p*
HR (mmHg)	81.65 ± 11.69	83.2 ± 13.08	0.188	73.08 ± 10.74	81.06 ± 12.86	**< 0.001**	78.23 ± 12.06	82.61 ± 13.03	**< 0.001**
PP (mmHg)	47.79 ± 8.13	50.18 ± 8.58	**0.002**	58.65 ± 8.86	51.38 ± 12.3	**< 0.001**	52.13 ± 9.96	50.53 ± 9.8	0.122
SBP (mmHg)	118.71 ± 10.75	120.36 ± 9.39	**0.019**	127.73 ± 10.43	122.36 ± 12.98	**0.001**	122.31 ± 11.49	120.94 ± 10.57	0.327
DBP (mmHg)	70.91 ± 7.48	70.81 ± 7.88	0.796	69.08 ± 7.98	71.35 ± 9.63	0.182	70.18 ± 7.73	70.96 ± 8.38	0.245
MAP (mmHg)	101.02 ± 16.05	96.83 ± 23.26	0.676	107.24 ± 13.29	104.31 ± 13.1	**0.02**	103.5 ± 15.3	98.89 ± 21.21	0.079

Values are presented as mean ± standard deviation (SD).

HR—heart rate, PP—pulse pressure, SBP—systolic blood pressure, DBP—diastolic blood pressure, MAP—mean arterial pressure.

*p*—values are indicated.

Significant values are in bold.

### Circulating inflammatory biomarkers

As the male and female participants recruited in Krakow and Lublin differed in terms of cardiovascular parameters, next we investigated whether there were differences in the circulating biomarkers between the groups. We found that Krakow residents had significantly higher levels of both C-reactive protein (CRP) and high sensitive C-reactive protein (hs-CRP), as well as fibrinogen and homocysteine in comparison to the subjects from Lublin (Table 3).

**Table 3:** Circulating inflammatory biomarkers in study participants from Krakow and Lublin.

Parameter	Krakow (n = 292)	Lublin (n = 284)	*p*
CRP (mg/l)	0.79 (0.56–1.20)	0.40 (0.20–0.70)	**< 0.001**
hs–CRP (mg/l)	0.52 (0.32–0.98)	0.35 (0.22–0.67)	**< 0.001**
Fibrinogen (mg/dl)	269.00 (226.50–311.70)	244.10 (210.20–267.90)	**< 0.001**
Homocysteine (µmol/l)	10.40 (9.13–12.15)	9.02 (7.69–10.80)	**< 0.001**

Values are presented as median (with an interquartile range).

CRP—C-reactive protein; hs-CRP—high sensitive C-reactive protein.

*p*—values are indicated.

Significant values are in bold.

These differences were observed in both female and male participants Table 5.

### Blood leukocyte subsets and biochemical parameters

We did not observe the differences in white blood cells (WBC) between young healthy subjects recruited in Kraków and Lublin. However, the analysis of the individual subpopulations revealed an increase in lymphocyte and monocyte counts in Lublin participants and especially females (Tables 4 and 5). Subjects from Krakow and especially females had significantly higher red blood cell (RBC) count, hematocrit (HCT), red cell distribution standard deviation (RDW-SD) and thyroid-stimulating hormone (TSH) than their Lublin colleagues (Tables 4 and 5). Moreover, both total cholesterol and high-density lipoprotein was significantly higher among Lublin subjects (Table 4). Male participants from Lublin had higher total cholesterol level than males from Krakow  (Table 5). Lublin participants both females and males had statically significant higher levels of mean corpuscular hemoglobin (MCH) and mean corpuscular hemoglobin concentration (MCHC) (Tables 4 and 5). The analysis of the concentration of uric acid (UA) revealed some differences between males and females as well the city of the residence (Table 5).

**Table 4:** Peripheral blood morphology, lipid panel, thyroid-stimulating hormone, glucose and uric acid in study participants from Krakow and Lublin.

Parameter	Krakow (n = 292)	Lublin (n = 284)	*p*
WBC (G/l)	6.29 ± 1.6	6.53 ± 1.66	0.058
LYMPH (G/l)	2.05 ± 0.52	2.17 ± 0.53	**0.005**
MONO (G/l)	0.55 ± 0.17	0.58 ± 0.16	**0.011**
NEUT (G/l)	3.49 ± 1.34	3.59 ± 1.41	0.28
EO (G/l)	0.18 ± 0.23	0.16 ± 0.18	0.29
BASO (G/l)	0.03 ± 0.02	0.03 ± 0.02	0.63
RBC (T/l)	4.92 ± 0.4	4.75 ± 0.41	**< 0.001**
Hb (g/dl)	14.16 ± 1.28	14.02 ± 1.25	0.19
HCT (%)	42.45 ± 3.13	40.99 ± 3.12	**< 0.001**
MCV (fl)	86.4 ± 3.59	86.38 ± 3.27	0.98
MCH (pg)	28.8 ± 1.46	29.52 ± 1.31	**< 0.001**
MCHC (g/dl)	33.34 ± 0.98	34.18 ± 0.9	**< 0.001**
RDW-SD (fl)	40.99 ± 2.59	39.6 ± 2.09	**< 0.001**
PLT (G/l)	249.75 ± 56.25	244.96 ± 52.79	0.49
CHOL (mg/dl)	159.79 ± 27.35	165.86 ± 27.67	**0.006**
TG (mg/dl)	78.23 ± 34.73	77.29 ± 36.70	0.42
LDL (mg/dl)	82.34 ± 22.91	84.24 ± 23.95	0.56
HDL (mg/dl)	61.73 ± 14.16	65.72 ± 15.67	**0.005**
TSH (mlU/l)	2.34 ± 1.14	2.07 ± 0.95	**0.007**
GLU (mg/dl)	86.58 ± 8.48	87.46 ± 7.41	0.10
UA (µmol/l)	291.4 ± 69.71	290.98 ± 66.82	0.97

Values are presented as mean ± standard deviation (SD).

WBC—white blood cells, LYMPH—lymphocytes, MONO—monocytes, NEUT—neutrophil, EO—eosinophil, BASO—basophil, RBC—red blood cells, HB—haemoglobin, HCT—haematocrit, MCH—mean corpuscular haemoglobin, MCHC—mean corpuscular haemoglobin concentration, RDW-SD—red blood cell distribution standard deviation, PLT—platelet , CHOL—total cholesterol, TG—triglycerides, LDL—low-density lipoprotein, HDL—high-density lipoprotein, TSH—thyroid stimulating hormone, GLU—glucose, UA—uric acid.

*p* values are indicated.

Significant values are in bold.

**Table 5:** Peripheral blood morphology, circulating inflammatory biomarkers, lipid panel, thyroid-stimulating hormone, glucose and uric acid in female and male participants from Krakow and Lublin.

	Female			Male		
Parameter	Krakow n = 176	Lublin n = 206	*p*	Krakow n = 116	Lublin n = 78	*p*
WBC (G/l)	6,42 ± 1,7	6,62 ± 1,73	0,181	6,1 ± 1,43	6,3 ± 1,46	0,213
LYMPH (G/l)	2,01 ± 0,51	2,15 ± 0,52	**0,008**	2,1 ± 0,54	2,23 ± 0,56	0,129
MONO (G/l)	0,53 ± 0,15	0,58 ± 0,16	**0,002**	0,58 ± 0,19	0,58 ± 0,16	0,834
NEUT (G/l)	3,68 ± 1,41	3,71 ± 1,5	0,846	3,2 ± 1,18	3,28 ± 1,07	0,389
EO (G/l)	0,17 ± 0,25	0,15 ± 0,18	0,957	0,2 ± 0,2	0,18 ± 0,17	0,363
BASO (G/l)	0,03 ± 0,02	0,03 ± 0,01	0,995	0,03 ± 0,01	0,03 ± 0,02	0,242
RBC (T/l)	4,7 ± 0,3	4,59 ± 0,28	**< 0,001**	5,26 ± 0,29	5,18 ± 0,4	0,596
Hb (g/dl)	13,39 ± 0,8	13,51 ± 0,8	0,133	15,34 ± 0,93	15,38 ± 1,22	0,147
HCT (%)	40,61 ± 2,08	39,74 ± 2,12	**< 0,001**	45,24 ± 2,27	44,29 ± 2,92	0,099
MCV (fl)	86,59 ± 3,76	86,65 ± 3,42	0,763	86,09 ± 3,3	85,65 ± 2,72	0,291
MCH (pg)	28,55 ± 1,5	29,45 ± 1,37	**< 0,001**	29,18 ± 1,31	29,71 ± 1,13	**0,004**
MCHC (g/dl)	32,97 ± 0,88	33,98 ± 0,81	**< 0,001**	33,89 ± 0,85	34,69 ± 0,94	**< 0,001**
RDW-SD (fl)	41,44 ± 2,71	39,8 ± 2,14	**< 0,001**	40,32 ± 2,23	39,07 ± 1,86	**< 0,001**
PLT (G/l)	260,31 ± 56,52	249,79 ± 52,19	0,103	233,72 ± 52,11	232,22 ± 52,57	0,905
CHOL (mg/dl)	163,57 ± 26,68	166,54 ± 27,91	0,294	154,05 ± 27,47	164,07 ± 27,15	**0,007**
TG (mg/dl)	75,05 ± 32,81	74,03 ± 34,38	0,553	83,01 ± 37,06	86,02 ± 41,28	0,793
LDL (mg/dl)	82,29 ± 21,99	82,22 ± 23,16	0,619	82,41 ± 24,35	89,55 ± 25,3	0,059
HDL (mg/dl)	66,21 ± 13,41	69,17 ± 15,36	0,083	54,9 ± 12,51	56,61 ± 12,58	0,451
TSH (mlU/l)	2,33 ± 1,11	2,03 ± 0,97	**0,004**	2,35 ± 1,19	2,17 ± 0,87	0,703
GLU (mg/dl)	85,94 ± 6,98	86,51 ± 6,68	0,269	87,56 ± 10,31	89,97 ± 8,6	**0,045**
UA (µmol/l)	289,22 ± 73,59	269,01 ± 50,6	**0,021**	294,64 ± 63,65	349,76 ± 69,67	**< 0,001**
CRP (mg/l)	0.79 (0,57–1.19)	0.4 (0.2–0.7)	**< 0.001**	0.78 (0.54–1.25	0.3 (0.2–0.7)	**< 0.001**
hs–CRP (mg/l)	0.52 (0.37–0.95)	0.36 (0.23–0.69)	**< 0.001**	0.54 (0.27–0.99)	0.31 (0.2–0.6)	**< 0.001**
Fibrinogen (mg/dl)	283 (237–322.6)	252.6 (224.7–276.8)	**< 0.001**	242.9 (208.4–280.5)	214.7 (183–252.93)	**< 0.001**
Homocysteine (µmol/l)	10 (8.48–11.17)	8.61 (7.52–10.1)	**< 0.001**	11.15 (9.5–12.9)	10.2 (8.83–11.8)	**0.019**

Values are presented as mean ± standard deviation (SD).

WBC—white blood cells, LYMPH—lymphocytes, MONO—monocytes, NEUT—neutrophil, EO—eosinophil, BASO—basophil, RBC—red blood cells, HB—haemoglobin, HCT—haematocrit, MCH—mean corpuscular haemoglobin, MCHC—mean corpuscular haemoglobin concentration, RDW-SD—red blood cell distribution standard deviation, PLT—platelet , CHOL—total cholesterol, TG—triglycerides, LDL—low-density lipoprotein, HDL—high-density lipoprotein, TSH—thyroid stimulating hormone, GLU—glucose, UA—uric acid, CRP—C-reactive protein; hs-CRP—high sensitive C-reactive protein.

*p*—values are indicated.

Significant values are in bold.

## Discussion

In this study we investigated the relationships between long-term exposure to different environmental factors and major cardiovascular parameters and inflammatory biomarkers in young, healthy subjects. The study participants were residents of two cities differing in air and noise pollutions. It is well known that the long-term exposures to environmental factors including airborne pollutants as well as noise are associated with cardiovascular risk^3,25^. In the present study, we have recruited young, healthy people. Interestingly, we found increased plasma level of inflammatory biomarkers such as hs-CRP and homocysteine in both male and female subjects from Kraków, in comparison to the inhabitants of Lublin. We did not observe any differences in both systolic and diastolic blood pressure between participants from Krakow and Lublin. Our observation is in line with previous studies that investigated the influence of air pollution and noise levels in children at the age of 10 years^26,27^. On the contrary to these findings, Bilenko et al. have found a positive association between DBP and long-term exposure to concentrations of air particulate matter in children at the age of 12 years^28^. Studies in adults are also inconsistent^29,30^. Fuks et al. have demonstrated the association of long-term exposure to PM with increased in both systolic and diastolic blood pressure in 4291 participants at the age of 45–75 years^29^. On the other hand, Sorensen et al. have provided the evidence that long-term exposure to traffic air pollution was not associated with hypertension^30^. The metanalysis of 17 studies suggested that long-term exposure to some air pollutants may increase risk of hypertension^31^.

Interestingly, we observed higher systolic blood pressure in males from Krakow in comparison to males recruited in Lublin. This observation is consistent with a study performed by Dong et al. who have shown that higher air pollution levels were associated with increased blood pressure in men^32^. Moreover, males from Krakow had significantly higher systolic blood pressure than females from both groups. On the contrary, females from Lublin had significantly higher SBP than females from Krakow. It cannot be excluded that males may have greater exposure to ambient air pollutants as they engage in more frequent and intense in outdoor activities^33^, which is a gender factor. Moreover, clinical data suggest that estrogen may have cardio-protective effects including renoprotection, prevention of vascular remodeling, sympathoinhibition and vasorelaxation^34^.

We also found that all subjects from Krakow, and particularly male participants had significantly lower heart rate than the corresponding groups from Lublin. Females living in Krakow had lower heart rate in comparison to females living in Lublin. However, it should be noted that this observation was not statistically significant. Our finding is consistent with previous studies showing an association between decreased heart rate variability, particularly through parasympathetic (vagal) modulation of cardiac autonomic function, and increased concentrations of the pollutants^35,36^.

Interestingly, we found that inflammatory biomarkers such as CRP, hs-CRP, fibrinogen and homocysteine were significantly elevated in Krakow cohort in comparison to Lublin subjects. CRP, hs-CRP, and fibrinogen are considered as acute phase proteins, which are increasing in response to both inflammation and injury. Furthermore, these biomarkers as well as homocysteine are positively associated with cardiovascular risk^37,38^. Activated by oxidative stress inflammatory cytokines may induce endothelial dysfunction and an imbalance in vascular homeostatic response. Furthermore, the high concentration of PM may induce vasoconstriction and an imbalance of the autonomic nervous system^31,39^. Interestingly, there is the relationship between inflammatory biomarkers, increased blood pressure and long term exposure to air pollution^40,41^. Elevated C-reactive protein was found in Japanese and Mexican children population exposed to suspended particulate matter (SPM)^42,43^. Shima et al. have found that an increase in CRP above 1.4 mg/L was significantly associated with mean levels of SPM^42^. To our knowledge we are the first to report the potential impact of air pollution on high sensitive CRP levels among young, healthy subjects. Importantly, hs-CRP is considered as a significant risk factor of subsequent cardiovascular events and death^44^. Our observation of increased hs-CRP level in population living in more polluted city is in line with study presented by Pilz and Lane^40,41^. It should be noted that their results were significant among white non-Hispanic population but not in general population. It is well known that increased level of fibrinogen is associated with the blood coagulation process even in healthy individuals^45^, which may explain the potential impact of air pollution as a risk factor for cardiovascular diseases. Rudez et al.^46^ have demonstrated that air pollution was associated with both increased thrombin generation and platelet aggregation but not with CRP and fibrinogen. However, it should be noted that the study group was older than ours.

Increased plasma homocysteine is associated with endothelial cell damage as well as reduction in vessels flexibility which lead to the atherosclerosis^47^. We found that homocysteine levels were significantly higher among Krakow population, both in men and women. This is consistent with previous published data^44^. Ren et al. have found that PM2.5 and black carbon were associated with elevated level of plasma homocysteine and this effect were modified by polymorphisms of GSTT1 and HFE C828Y genes^48^.

Recently published paper has suggested that the noise may also have negative effect on cardiovascular system^1^. On the other hand according to Kuntic et al. noise and air pollution have additional adverse effects on the cardiovascular system^49^. Interestingly, in our study, the noise level was greater in Lublin as compared to Krakow. However, it should be noted that the different environmental factors may vary even in the same area resulting in the additional burden. Our results may suggest that some of the environmental factors have greater influence on cardiovascular system than others. Similarly, the ESCAPE study revealed that air pollution was the major risk factor, opposed to the noise levels^50^. Andersson et al. have shown that the noise levels above 60 db could have negative influence on cardiovascular system as compared to noise levels lower than 50 db in the group of 6304 participants^51^. Importantly, both in Krakow and Lublin only minority of inhabitants were exposed to noise levels over 60 db. Further research is needed to demonstrate how these factors could affect cardiovascular risk as well as the expression of circulating inflammatory biomarkers. Our study suggests that air pollution may be one of the main factors influencing cardiovascular outcomes, in contrast to the noise level. Worth to acknowledge is fact that according to Lelieveld et al. study conclusions ‘ambient air pollution would no longer be a leading, environmental health risk factor if the use of fossil fuels were superseded by equitable access to clean sources of renewable energy’^52^.

### Study limitations

We compared residents of two cities, where the degree of exposure to specific factors, such as air pollution and noise was different. On this basis, we conclude that the place of inhabitance may have a significant impact on inflammatory and cardiovascular parameters. Nevertheless, it was not possible to assess the direct effects of the individual pollutants such PM2.5 or PM10 concentration or noise levels on observed phenomena.

We are aware of the potential presence of additional factors for example the exposition to mold, dampness, stress level, physical activity, nutritional profile and eating habits, which have not been studied. Also, the effect of exposures to indoor air in residences could not be assessed. Finally, the number of patients was relatively small. It should be noted that the surveys concerned only simple records on personal data, symptoms lifestyle, pro-health behavior and the living in the city for at least 10 years. Another limitation may be the bias in blood pressure measurements.

## Conclusions

To conclude, our study suggests that long-term exposure to environmental factors associated with place of inhabitance may have significant impact on cardiovascular parameters and level of inflammatory biomarkers in young, healthy subjects. Moreover, the differences in blood pressure showed significant sexual dimorphism.

### Supplementary Information

Supplementary Table S1.

## Data Availability

The datasets used and/or analyzed during the current study are available from the corresponding author upon reasonable request.
