# Supplementary material for: Sex-specific associations between the environmental exposures and low-grade inflammation and increased blood pressure in young, healthy subjects
Source: Sci Rep. 2024 Apr 26;14:9588. doi: 10.1038/s41598-024-59078-4 (PMC11053153; doi:10.1038/s41598-024-59078-4)
Supplement: Supplementary file 1 — Supplementary Table S1. [file 41598_2024_59078_MOESM1_ESM.docx]

# Supplementary table

**Table S1.** Peripheral blood morphology, lipid panel, thyroid-stimulating hormone, glucose, uric acid and circulating inflammatory biomarkers in study participants from Krakow and Lublin.

| Parameter | Krakow (n=292) | Lublin (n=284) | p | Reference values |
| --- | --- | --- | --- | --- |
| WBC [G/l] | 6.29±1.6 | 6.53±1.66 | 0.058 | 3.50 - 10.00 |
| LYMPH [G/l] | 2.05±0.52 | 2.17±0.53 | **0.005** | 1.00 - 5.00 |
| MONO [G/l] | 0.55±0.17 | 0.58±0.16 | **0.011** | < 0.80 |
| NEUT [G/l] | 3.49±1.34 | 3.59±1.41 | 0.28 | 1.80 - 7.70 |
| EO [G/l] | 0.18±0.23 | 0.16±0.18 | 0.29 | < 0.45 |
| BASO [G/l] | 0.03±0.02 | 0.03±0.02 | 0.63 | < 0.20 |
| RBC [T/l] | 4.92±0.4 | 4.75±0.41 | **<0.001** | 3.50 - 6.50 |
| Hb [g/dl] | 14.16±1.28 | 14.02±1.25 | 0.19 | 12.00 - 16.80 |
| HCT [%] | 42.45±3.13 | 40.99±3.12 | **<0.001** | 40.00 - 49.50 |
| MCV [fl] | 86.4±3.59 | 86.38±3.27 | 0.98 | 82.00 - 103.00 |
| MCH [pg] | 28.8±1.46 | 29.52±1.31 | **<0.001** | 27.00 - 34.00 |
| MCHC [g/dl] | 33.34±0.98 | 34.18±0.9 | **<0.001** | 32.00 - 36.00 |
| RDW-SD [fl] | 40.99±2.59 | 39.6±2.09 | **<0.001** | 37.00 - 54.00 |
| PLT [G/l] | 249.75±56.25 | 244.96±52.79 | 0.49 | 125.00 - 400.00 |
| CHOL [mg/dl] | 159.79 ± 27.35 | 165.86 ± 27.67 | **0.006** | < 190.00 |
| TG [mg/dl] | 78.23 ± 34.73 | 77.29 ± 36.70 | 0.42 | < 150.00 |
| LDL [mg/dl] | 82.34 ± 22.91 | 84.24 ± 23.95 | 0.56 | < 100.00 |
| HDL [mg/dl] | 61.73 ± 14.16 | 65.72 ± 15.67 | **0.005** | > 40.00 |
| TSH [mlU/l] | 2.34 ± 1.14 | 2.07 ± 0.95 | **0.007** | 0.27 - 4.20 |
| GLU [mg/dl] | 86.58±8.48 | 87.46±7.41 | 0.10 | 3.90 - 5.50 |
| UA [µmol/l] | 291.4±69.71 | 290.98±66.82 | 0.97 | 202.00 - 410.00 |
| CRP [mg/l] | 0.79 (0.56 - 1.20) | 0.40 (0.20 - 0.70) | **< 0.001** | < 5.00 |
| hs–CRP [mg/l] | 0.52 (0.32 - 0.98) | 0.35 (0.22 - 0.67) | **< 0.001** | < 1.00 |
| Fibrinogen [mg/dl] | 269.00 (226.50 - 311.70) | 244.10 (210.20 - 267.90) | **< 0.001** | 200.00 - 400.00 |
| Homocysteine [µmol/l] | 10.40 (9.13 - 12.15) | 9.02 (7.69 - 10.80) | **< 0.001** | < 12.00 |

Values are presented as mean ± standard deviation (SD) or median (with an interquartile range).

WBC – white blood cells, LYMPH – lymphocytes, MONO – monocytes, NEUT – neutrophil, EO – eosinophil, BASO – basophil, RBC – red blood cells, HB – haemoglobin, HCT – haematocrit, MCH – mean corpuscular haemoglobin, MCHC – mean corpuscular haemoglobin concentration, RDW-SD – red blood cell distribution standard deviation, PLT – platelet , CHOL – total cholesterol, TG – triglycerides, LDL – low-density lipoprotein, HDL – high-density lipoprotein, TSH – thyroid stimulating hormone, GLU – glucose, UA – uric acid, CRP – C-reactive protein; hs-CRP – high sensitive C-reactive protein, p values and reference values are indicated.
